# Changes in Lactate Production, Lactate Dehydrogenase Genes Expression and DNA Methylation in Response to Tamoxifen Resistance Development in MCF-7 Cell Line

**DOI:** 10.3390/genes12050777

**Published:** 2021-05-19

**Authors:** Lama Hamadneh, Lara Al-Lakkis, Ala A. Alhusban, Shahd Tarawneh, Bashaer Abu-Irmaileh, Sokiyna Albustanji, Abdel Qader Al-Bawab

**Affiliations:** 1 Faculty of Pharmacy, Al-Zaytoonah University of Jordan, Amman 11733, Jordan; lara.lakkiis@gmail.com (L.A.-L.); ala.alhusban@zuj.edu.jo (A.A.A.); sukainabustanji94@gmail.com (S.A.); abdelqader.albawab@zuj.edu.jo (A.Q.A.-B.); 2 Faculty of Science, Mutah University, Karak 61710, Jordan; shahdtarawneh0@gmail.com; 3 Hamdi Mango Center for Scientific Research, The University of Jordan, Amman 11942, Jordan; bashaeraburmaileh@yahoo.com

**Keywords:** *LDHB* hypomethylation, breast cancer, lactate, *LDHA* and *LDHB* gene expression, tamoxifen resistance

## Abstract

Lactate dehydrogenase (LDH) is a key enzyme in the last step of glycolysis, playing a role in the pyruvate-to-lactate reaction. It is associated with the prognosis and metastasis of many cancers, including breast cancer. In this study, we investigated the changes in *LDH* gene expression and lactate concentrations in the culture media during tamoxifen resistance development in the MCF-7 cell line, and examined *LDHB* promoter methylation levels. An upregulation of 2.9 times of *LDHB* gene expression was observed around the IC50 concentration of tamoxifen in treated cells, while fluctuation in *LDHA* gene expression levels was found. Furthermore, morphological changes in the cell shape accompanied the changes in gene expression. Bisulfate treatment followed by sequencing of the *LDHB* promoter was performed to track any change in methylation levels; hypomethylation of CpG areas was found, suggesting that gene expression upregulation could be due to methylation level changes. Changes in *LDHA* and *LDHB* gene expression were correlated with the increase in lactate concentration in the culture media of treated MCF-7 cells.

## 1. Introduction

Breast cancer remains a major health problem in most parts of the world, despite the advances achieved in the field [1]. Female breast cancer incidence has exceeded lung cancer as the most common cancer in 2020, with an estimated 2.3 million new cases [2]. Although the mortality rate for women with an already confirmed diagnosis has been declining [3], it remains the leading cause of cancer deaths among women [2].

Tamoxifen treatment in estrogen receptor-positive patients reduced recurrence up to 9 years after acquiring cancer. Also, breast cancer mortality rates were significantly reduced by about one third through the first 15 years of follow up among tamoxifen treated patients [4,5]. However, even in the presence of many therapeutic options, drug resistance remains a challenging issue in cancer treatment, as approximately a quarter of breast cancer cases treated with tamoxifen for 5 years displayed tamoxifen resistance (TamR) [4,6].

The mechanisms underlying tamoxifen resistance are complex and many of them remain unknown [7]. The alteration of gene expression and signaling pathways was reported to have a role in inducing TamR [8], in which, significant degradation of estrogen receptors (ER) was noticed in TamR cancer cells [9]. Additionally, activation of the mitogen-activated protein kinase (*MAPK*) signaling pathway and the phosphatidylinositol 3-kinase/protein kinase B (*PI3K/AKT*) pathway have been reported to have roles in cell proliferation, regrowth, autophagy, and endocrine resistance [10,11].

Many cancer cells convert most of the pyruvate to lactate, whether there is oxygen or not, in a phenomenon called the Warburg effect [12]. In breast cancer, lactate is produced mainly by the activity of lactate dehydrogenase A (*LDHA*), and it was studied to be used as a predictive marker for prognosis and overall survival in patients [13]. On the other hand, some studies reported that lactate dehydrogenase B (*LDHB*) gene expression was found to be reduced in many commonly used breast cancer cell lines due to the hypermethylation of the promoter area leading to gene silencing [14], while other researchers reported that upregulated gene and protein expression are seen in triple-negative cells in comparison to luminal breast cancer cells [15].

In this study, lactate dehydrogenase A and B gene expression levels were determined during tamoxifen resistance development in MCF-7 cell lines and correlated with the concentration of lactate secreted to the culture media.

## 2. Materials and Methods

Cell culturing and tamoxifen treatment

MCF-7 (HTB-22™) cells (ATCC, Manassas, VA, USA) of passage 9 were cultured in RPMI medium (EuroClone S.p.A., Via Figino, Italy) containing 10% fetal bovine serum (FBS), 1% penicillin–streptomycin, and sodium pyruvate (EuroClone S.p.A., Via Figino, Italy). Cells were grown in a humidified incubator under 5% CO_2_ at 37 °C. Growth medium was routinely replaced. When cells were 70% confluent, they were treated with low concentrations of tamoxifen starting with a concentration of 10 nM, incubated for 3 days, then fresh media with no tamoxifen was added and the cells were allowed to grow until 70% confluency before the next tamoxifen concentration was added. Tamoxifen concentrations were gradually increased up to 40 µM to induce resistance.

Gene expression and DNA methylation analysis

DNA and RNA from treated cells were extracted using the innuPREP DNA/RNA Mini Kit (Analytik Jena, Jena, Germany) according to the manufacturer’s protocols. DNA and RNA were quantified to be used in DNA methylation and gene expression analysis, respectively.

After quantification and a PCR integrity check, total mRNA samples were used to synthesize cDNA using the SuperScript^®^ VILO™ cDNA Synthesis Kit (Life Technologies, Grand Island, NY, USA), and gene expression analysis of lactate dehydrogenase A and lactate dehydrogenase B was performed using the following primers:

*LDHA* F: 5′ CTCTGGCAAAGTGGATATCTTGAC 3′ and

R: 5′ GGTAACGGAATCGGCTGAA 3′;

*LDHB* F: 5′ CTCTCCTGGTAGGTTTCGGC 3′ and

R: 5′ GCCGGATGCTCAGAGCTAAA 3′.

DNA methylation analysis:

DNA samples were bisulfate-treated using the EZ DNA Methylation-Gold Kit (ZYMO Research Corp., Irvine, CA, USA) according to the kit’s protocols. Treated DNA samples were used to determine the DNA methylation levels of lactate dehydrogenase B promotor. PCR amplification followed by sequencing was performed using two sets of primers to sequence *LDHB* promoter (set 1 and set 2). The sequences of set 1 were previously used by Leiblich et al. [16] and gave 197bp by PCR with 14 CpG sites, while set 2 was used by Maekawa et al. [17] and gave 282bp with 14 CpG sites. All primers were ordered from Integrated DNA Technologies, Inc. (IDT, Coralville, IA, USA)

Set 1 F: 5′ TTTGGTTTATAGGTAAGTTTGATGG 3′ and

R: 5′ ACTACTACCCTCTACCTTCTACTCCTC 3′;

Set 2 F: 5′ AGGGAGTGTGTATATTTGAGTT 3′ and

R: 5′ TCAAACTTACCTATAAACCAAA 3′.

Lactate detection using capillary electrophoresis—conductivity detector (CE-C^4^D):

The supernatant media from treated MCF-7 cells were collected, and 1 mL was transferred into 2 mL of Milli-Q water in a glass vial to form a diluted working solution with 1:2 in ratio and mixed very well, then filtered with syringe filters of 45 µM pore size. The analysis operation was performed using the optimized method of an in-house built CE-C^4^D as in [18,19] at room temperature, and the flow rate was also optimized for best analysis to separate each peak of analytes without overlapping or broadening.

The standard solution of lactate (Sigma-Aldrich, St. Louis, MO, USA) was prepared by dissolving the powdered lactate in 10 mL of Milli-Q water to prepare 200 mM. Then, a serial dilution was applied to prepare the working solutions with different concentrations of lactate as follows: 0, 0.5, 1 and 2 mM.

## 3. Results

**Figure 1:**
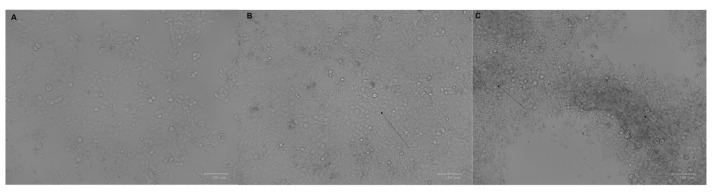
Morphological changes accompanied tamoxifen resistance development; (**A**) untreated control MCF-7 cells, (**B**) MCF-7 cells treated with 30 µM tamoxifen and (**C**) MCF-7 cells treated with 40 µM tamoxifen. Arrows indicate the change in cell shape in comparison to the epithelial-like shape of MCF-7 cells. Images were taken using ZOE Fluorescent Cell Imager (Bio-Rad, Hercules, CA USA) (scale bar 100 μm). Experiments were repeated at 3 different times.

Morphological changes of breast cancer cell line MCF-7 were observed during the process of tamoxifen resistance development as reported previously [20] and are shown in Figure 1. Cells treated with 30 µM tamoxifen started to lose their epithelial-like shape and became round. At concentration 40 µM, the cells started to aggregate, and cells’ rate of growth increased.

**Figure 2:**
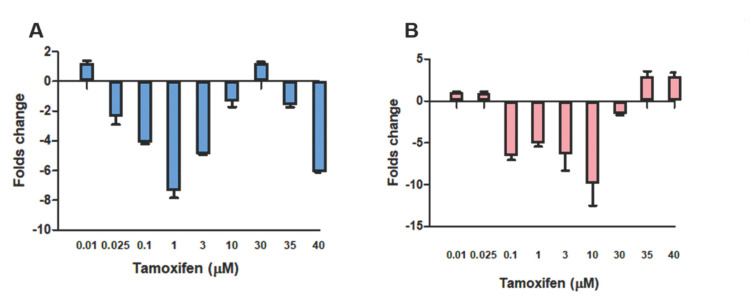
Fold changes in gene expression of (**A**) *LDHA* and (**B**) *LDHB* from MCF-7 cells treated with increasing doses of tamoxifen. Gene expression concentration results were expressed as mean ± SD (*n* = 3 runs for each sample).

**Figure 3:**
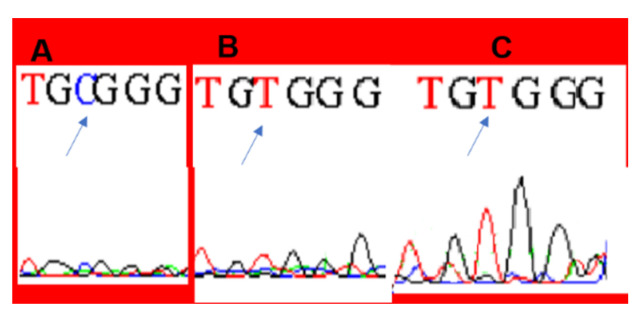
Promoter hypomethylation seen in bisulfate-treated DNA samples from cells treated with (**B**) 35 and (**C**) 40 µM tamoxifen in comparison with untreated control cells (**A**). Unmethylated cytosine was replaced with thymine after bisulfate treatment in cells treated with 35 and 40 µM tamoxifen.

Gene expression analysis of *LDHA* and *LDHB* is presented in Figure 2. *LDHA* gene expression showed fluctuation but was mostly downregulated with increased tamoxifen doses. *LDHB* downregulation was maintained until 30 µM (−1.6), after which a significant change was seen in MCF-7 cells treated with 35 µM, and the upregulation was maintained in cells treated with 40 µM, where the fold change was 2.9 in both treated cells. These changes in *LDHB* gene expression were accompanied by promoter hypomethylation in these cells after sequencing of bisulfate-treated DNA samples (Figure S1). Promoter hypomethylation was observed as seen in Figure 3.

**Figure 4:**
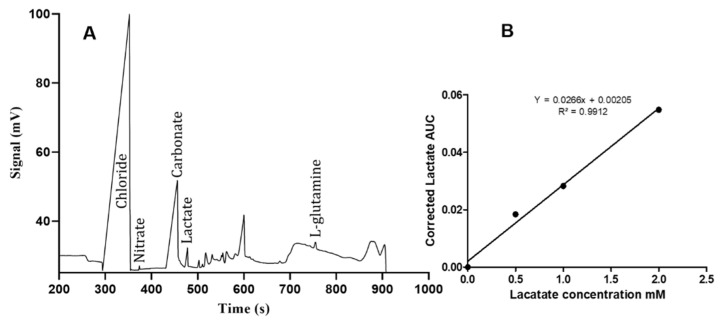
(**A**) Electropherogram of analyzed anions in prepared RPMI-1640 cell culture media. Conditions were as follow: 90 cm × 50 μm I.D. fused silica capillary coated with HDMB/PSS/HDMB. BGE: 30 mM TRIS/30 mM CHES, pH 8.4 with 0.025% PEI; +30 kV applied to outlet vial while interface was grounded. Signal was obtained using a Trace DEC conductivity detector C^4^D positioned 10 cm from the outlet. (**B**) Calibration curve of lactate by CE-C4D, using 4 different concentrations.

**Figure 5:**
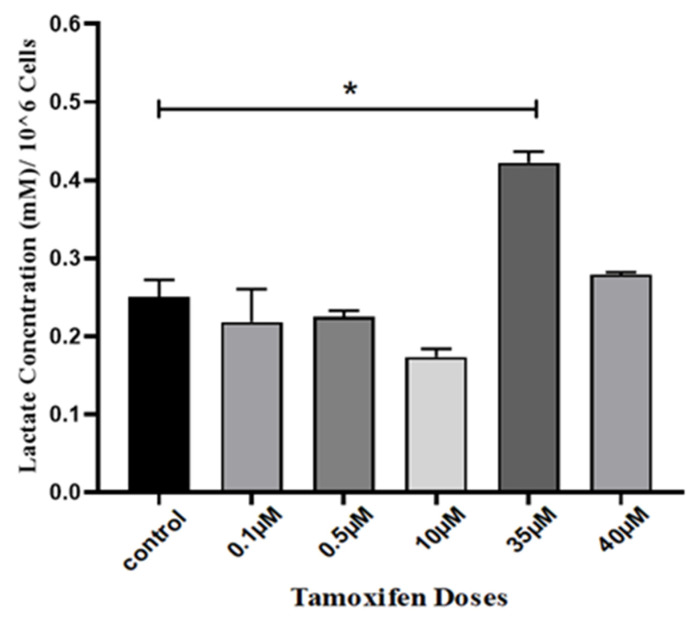
Calculated concentration by CE-C4D of produced lactate from MCF-7 cell supernatant media normalized with cell density after treatment with tamoxifen in gradual increased doses. Statistical significance was calculated by one-way ANOVA followed by Tukey post hoc test in GraphPad prism 8.0 software, considering the statistical significance as follows: * significant at *P* ≤ 0.05; results were expressed as mean ± SD (*n* = 3 runs for each sample).

To detect the changes in lactate concentrations during tamoxifen resistance development in MCF-7 cells, the electrophoretic analysis was prepared for RPMI-1640 media alone as presented in Figure 4A. The calibration curve of lactate by CE was calculated by spiking RPMI-1640 media with four different concentrations of lactate (0, 0.5, 1 and 2 mM), then the AUC of lactate peaks were calculated and corrected with chloride peaks as an internal standard, as shown in Figure 4B. Based on the calculated equation from the calibration curve, the analysis was made for the developed tamoxifen-resistant MCF-7 cells, and the lactate concentration was measured in the acquired supernatant media. The concentration of lactate was measured from the AUC in electropherograms after correction with the chloride AUC as an internal standard, as shown in Figure 5.

## 4. Discussion

Breast cancer cells’ production of lactate under aerobic conditions contribute to their proliferation, angiogenesis, and aggressive behavior [21]. Lactate was also found to have an oncometabolic effect, where a transcriptional increase in the PIK3/AKT/mTOR signaling pathway accompanying lactate exposure in MCF-7 cells was seen [22].

The role of lactate dehydrogenases in lactate metabolism has been studied extensively in breast cancer, and many have reported that *LDHA* overexpression is seen under hypoxic conditions and is associated with c-*MYC* gene overexpression and glutaminolysis [23]. It was also found to be overexpressed in Taxol-resistant breast cancer cells [24]. On the other hand, *LDHB* was reported to be silenced in the estrogen and progesterone-positive MCF-7 cell line due to promoter hypermethylation [14,25]. However, *LDHB* was found to be overexpressed in triple-negative breast cancer cell lines and was correlated with poor prognosis among breast cancer patients [15]; it was also found to be overexpressed in highly glycolytic, mesenchymal breast cancer cell lines [26].

In this study, we correlated the changes in lactate levels in MCF-7 culture media with gene expression of *LDHA* and *LDHB* during the process of tamoxifen resistance development. Gradual increase in TAM doses was accompanied with downregulation of *LDHA* gene expression and a significant increase in *LDHB* gene expression, parallel with demethylation of certain CpG sites in the promoter region. Interestingly, these changes in *LDHA* and *LDHB* gene expression were in parallel with our reported finding that there was no *c-MYC* gene overexpression, with a significant increase in glutamine production during TamR development [20].

Different methods of tamoxifen resistance development in the MCF-7 cell line could affect gene expression differently [20]. Using the gradual increase in tamoxifen concentrations reported in this study, we showed that the increase in *LDHB* gene expression but not *LDHA* was correlated with lactate concentration increase in the media during the process of TamR development.

Lactate overproduction is linked to the Warburg effect in different types of cancer cells. The Warburg effect is also more favorable in these cells than oxidative phosphorylation for energy production, despite the presence of an adequate level of oxygen, and consequently results in the conversion of pyruvate into lactate [27]. Accumulation of lactate in the tumor cell microenvironment was reported to have a key role in carcinogenesis and tumor invasion [28] and could serve as a possible resistance biomarker and drug target.

## 5. Conclusions

Lactate participation in tumorigenesis is well documented and its role as a metabolic marker and therapeutic target has been explored, with most reports focused on the role of *LDHA* in breast cancer resistance to treatment, and few focused on *LDHB*. In this study, we report the potential involvement of lactate dehydrogenase B in breast cancer resistance to treatment that could provide a molecular marker to detect early resistance to tamoxifen among patients.

## Data Availability

Not applicable.

## Supplementary Materials

The following are available online at https://www.mdpi.com/article/10.3390/genes12050777/s1, Figure S1: DNA sequencing of bisulfate-treated samples file.
