# Supplementary material for: Changes in Lactate Production, Lactate Dehydrogenase Genes Expression and DNA Methylation in Response to Tamoxifen Resistance Development in MCF-7 Cell Line
Source: Genes (Basel). 2021 May 19;12(5):777. doi: 10.3390/genes12050777 (PMC8160872; doi:10.3390/genes12050777)

File: B2_B.ab1   *Run Ended: 2018/7/27 21:55:0*   *Signal G:136 A:367 C:77 T:504*

Sample: B2_B   *Lane: 10*   *Base spacing: -15.016356*   *393 bases in 9534 scans*

10 20 30 40 50 60 70 80 90 100 110 120

C CCCC C GATT CGGTTT GGAT TAAG GTCA C TATA A ACA G T T A GGATAG AATGG AATTTA GATTTTA GTT AT GTT AA TTTTA GTGTT GGTTTATA CTGAATTTT GAATAAATT AAT AAGAAATTA GTG

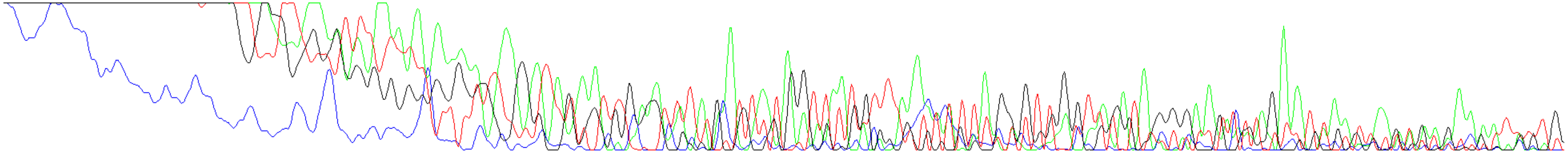

130 140 150 160 170 180 190 200 210 220 230 240 250

AGTTTA GTAATTATAT GGATT CTT GTTAAAT GATTATTTA CT AGGTTTATTGTTAAATTT GAAAAGAGTTTATT GTTTT GCGATTTTTAAATTATATTAGAATTTGTAGG CACCTAAA CGGCTCG

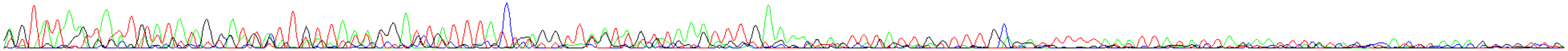

260 270 280 290 300 310 320 330 340 350 360

GTTTACAGGCACG TT CT AG CCC CGGACGCTC C GACCCCG ACC CATGC GGC GT G C AGCC GC GG C A TCC CCGG GCC C A CC C GC CCTCCCAG CC TC G AGAAC ACCCCG CC ACCG CTGC AC

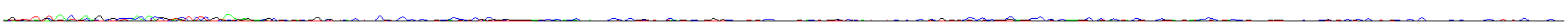

370 380 390

A GT GCCG CC AGCTTT CC CC C CC AGG

File: B2_B.ab1 Run Ended: 2018/7/27 21:55:0 Signal G:136 A:367 C:77 T:504

Sample: B2_B Lane: 10 Base spacing: -15.016356 393 bases in 9534 scans Page 2 of 2

File: B9_B.ab1 *Run Ended: 2018/7/27 21:55:0* *Signal G:491 A:624 C:477 T:550*

Sample: B9_B *Lane: 27* *Base spacing: 13.90427* *293 bases in 3733 scans*

10 20 30 40 50 60 70 80 90 100

TAATGAGTCCCGCTTAGGTATGATTCGTTAATTTAATCCCGTAGTGGGGACTATTGATAGAGTACGCTTTCGCGCTCTTTTTGAGGAGACCAACGAATCTTTTTGAAAC

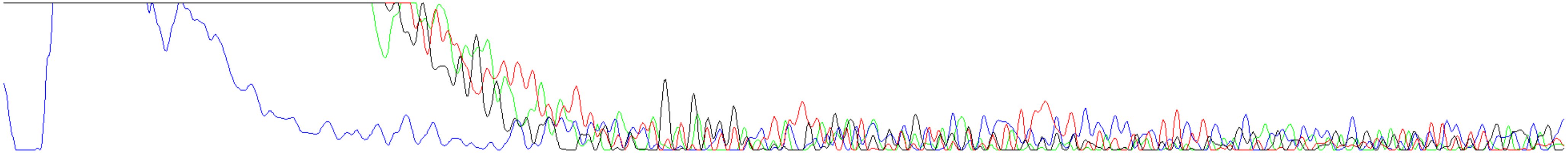

110 120 130 140 150 160 170 180 190 200 210 220 230

ACTATCTACCGCGCCATCATCATCCTAGCAGCAAAAACTCGGAACCACCCTGGTGCTTGACGTCATTACAAAGATCCACGCGGGAAGTTATGCTGATCATCGTTTATAATAACTCCCCACCAC

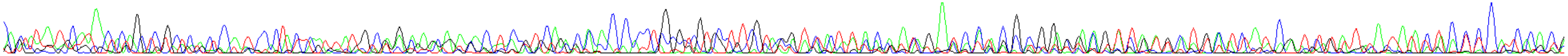

240 250 260 270 280 290

GACGACTCTCTATCAAAAGTCGATTCCCCCCTTGTGGACGCACTCAAACCCCCCAAACAAA

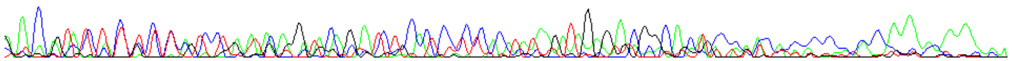

File: B10_B.ab1   Run Ended: 2018/7/27 21:55:0   Signal G:410 A:497 C:235 T:457

Sample: B10_B   Lane: 25   Base spacing: 13.868502   341 bases in 4332 scans   Page 1 of 1

10 20 30 40 50 60 70 80 90 100

CACGATCACCTGACTCATTTGACGTAGAAGTTCGCACACCTCGAAGGGATTTTGGCCCCGTACCCGACTCCCCTTTTTAACGCAGACCCGGCTATCTTTTTGAAATAC

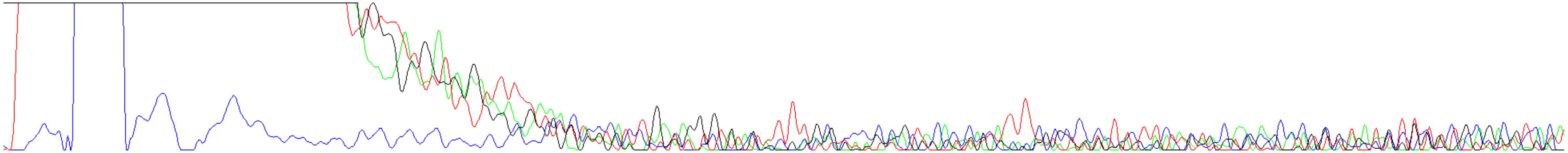

110 120 130 140 150 160 170 180 190 200 210 220 230

TACCAACTGTTCCGTCATCTTTTTTGCAAAACGGCTATTAACCACCTTGGATCTTGTAATACTACCATTATCCGGGTGGGGCGTTAAGATGATCATCGTTTTGACAACTCTCCATAACGACAA

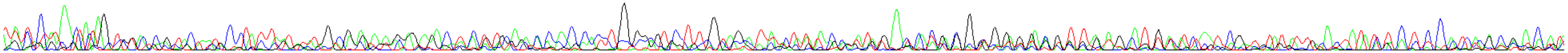

240 250 260 270 280 290 300 310 320 330 340

TTCCCTACCGAAAGTCGATTCCCTTAAAAGTGGACCCAATTTCAAATCCCAAAAAAAAACCCTCCTATTATTTAGAAGTGAACCTTTCATCTTTTGCCTTTGTGGGCAAG

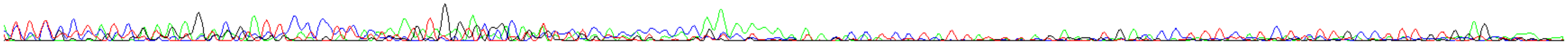

Supplement: Supplementary file 1 [file genes-12-00777-s001.zip › genes-1178765-supplementary.pdf]
